# Adipokine Retinol Binding Protein 4 and Cardiovascular Diseases

**DOI:** 10.3389/fphys.2022.856298

**Published:** 2022-03-02

**Authors:** Yanjing Ji, Jinyou Song, Tianhong Su, Xiaosong Gu

**Affiliations:** Department of Cardiology, The Second Affiliated Hospital of Soochow University, Suzhou, China

**Keywords:** retinol-binding protein 4, cardiovascular disease, lipids metabolism, vascular injury, adipokine

## Abstract

The morbidity and mortality of cardiovascular diseases (CVDs) have been increasing year by year all over the world and expanding greatly to the younger population, which becomes the leading causes of death globally that threatens human life safety. Prediction of the occurrence of diseases by using risk related adverse events is crucial for screening and early detection of CVDs. Thus, the discovery of new biomarkers that related to risks of CVDs are of urgent in the field. Retinol-binding protein 4 (RBP4) is a 21-kDa adipokine, mainly secreted by adipocytes. Besides its well-established function in the induction of insulin resistance, it has also been found in recent years to be closely associated with CVDs and other risk factors, such as hypertension, coronary heart disease, heart failure, obesity, and hyperlipidemia. In this review, we mainly focus on the progress of research that establishes the correlation between RBP4 and CVDs and the corresponding major risk factors in recent years.

## Introduction

Retinol-binding protein 4 (RBP4), whose gene is located near chromosome 10 (10q23-q24), is a 21-kDa protein secreted by hepatocytes and adipocytes. RBP4, as the sole carrier of retinol in the blood, increases the hydrophilicity of retinol upon binding and completes the transport of retinol from the liver to target tissues. Retinol-bound RBP4 (HoloRBP) further complexes with the tetrameric structure of transthyretin protein (TTR) to form a retinol transport complex, which prevents glomerular filtration of HoloRBP (Hamilton and Benson, 2001) and effectively increases and maintains the circulating concentration of RBP4. Initially, RBP4 was found to be involved in the pathogenesis of insulin resistance in type 2 diabetic patients (Yang et al., 2005). In recent years, more studies have suggested that RBP4 is also closely associated with lipid parameters and cardiovascular disease (Broch et al., 2010).

Multiple factors have now been found to influence circulating RBP4 concentrations. Adipose tissue is not only an energy-preserving tissue but can also release numerous substances known as “adipokines” or “adipocytokines.” Adipocytes are the main source of RBP4 secretion, Atrial natriuretic peptide (ANP) directly regulates the secretory activity of adipocytes in adipose tissue (Moro et al., 2007) and reduces the production of RBP4. RBP4 is excreted mainly from the kidneys. In patients with type 2 diabetes, microalbuminuria and glomerular filtration rate (GFR) are independent determinants of elevated serum RBP4 levels (Akbay et al., 2010). But it was shown that RBP4 is already diagnostically elevated before their appearance (Abbasi et al., 2020).

There are other factors regulate the serum RPB4. The effect of exercise on RBP4 levels depends on the intensity of exercise (Yu et al., 2009), with high levels of physical activity significantly reducing circulating RBP4 concentrations, but moderate and lower intensity activities have no significant effect on RBP4 concentrations. Resistance exercise reduces circulating RBP4 levels without altering intramuscular adipocytes or insulin resistance (Ku et al., 2010), whereas neither anaerobic exercise nor controls reduce RBP4. One mechanism for the effect of exercise on RBP4 may be an elevation of ANP (Niessner et al., 2003). The effect of diet on the magnitude of the decrease in circulating RBP4 depends on the amount of weight loss and the nature of the food, with carbohydrate-restricted diets leading to a greater decrease in serum RBP4 levels compared to low-fat diets (Volek et al., 2009). Statins do not seem to produce a significant effect on RBP4 (Szendroedi et al., 2009). The effect of glucose-lowering drugs on RBP4 concentrations in diabetic or non-diabetic patients has not been consistently concluded (Yao-Borengasser et al., 2007; Lin et al., 2008; Pfützner et al., 2009).

In recent years RBP4 have achieved significant efficacy coronary heart disease, hypertension, heart failure (Zhang et al., 2021). In this review, we will focus on RBP4 and their implication in cardiovascular disorder. The purpose of this review is to summarize current information on the RBP4 and risk factors of CVD.

### Retinol-Binding Protein 4 and Lipids Metabolism

Abnormal lipid metabolism is the most important risk factor for atherosclerosis, and hyperlipidemia, which includes hypercholesterolemia, hypertriglyceridemia, or both, requires binding to apolipoprotein plasm in plasma in the form of lipoproteins due to its lipid-soluble physical properties. The expression of RBP4 is negatively correlated with blood cholesterol (TC) levels (Jugnam-Ang et al., 2015). and the underlying mechanism may be that hypercholesterolemia causes adipocyte cholesterol overload, which interferes with adipocyte differentiation and maturation, causing adipocyte hypertrophy, adipose tissue inflammation (Mohapatra et al., 2011; Aguilar and Fernandez, 2014), and endocrine dysfunction, and adipose inflammation can lead to the release of pro-inflammatory factors (for example, TNF-α, IL-1β), and the accumulation of pro-inflammatory factors further inhibits the release of RBP4 from adipocytes into the blood (Zoccali et al., 2003).

Usui et al. (2009) found that RBP4 was positively associated with small and dense low-density lipoprotein (sdLDL) levels in young women and RBP4 was one of major factors affecting sdLDL-cholesterol levels. Similarly, sdLDL was found to be an independent predictor of oxidized low-density lipoprotein (ox-LDL) in patients with dyslipidemia, and sdLDL may be an important link between RBP4 and ox-LDL (Wu et al., 2012). It is now known that sdLDL and ox-LDL are components of atherogenic lipoproteins, and RBP4 may be involved in atherogenesis by directly or indirectly upregulating sdLDL levels. SdLDL was found to be an independent predictor of RBP4 in patients with dyslipidemia (Wu et al., 2012). Also, blood RBP4 levels were found to be negatively associated with indirect VLDL-apoB100 FCR and not significantly associated with direct VLDL-apoB100 FCR, suggesting that RBP4 is associated with more with VLDL dilapidation compared to direct uptake (Vergès et al., 2012), which may also explain the relationship between RBP4 and blood triglycerides in patients with type 2 diabetes. Wessel et al. (2019) found that RBP4 levels were positively correlated with large very low-density lipoproteins (VLDL) versus small LDL, but no physical interaction was found between them. In patients with type 2 diabetes, RBP4 has a strong positive correlation with blood triglyceride (TG) levels (Vergès et al., 2012; Wessel et al., 2019; Table 1), The same correlation with blood triglycerides and HDL was found in RBP4 single nucleotide polymorphisms (Codoñer-Franch et al., 2016). All these results suggest that RBP4 may be involved in the pathophysiological process of atherosclerosis by altering the distribution of proatherosclerotic plasma lipoproteins.

**TABLE 1:** Associated factors and diseases correlated with RBP4 levels.

	Involved diseases	Correlation with blood RBP4 levels	References
ANP	Heart failure	Negative	Moro et al., 2007
GFR	Chronic kidney disease	Negative	Akbay et al., 2010; Abbasi et al., 2020
High-intensity exercise	—	Negative	Yu et al., 2009
Blood cholesterol	Abnormal lipid metabolism, T2DM	Negative	Jugnam-Ang et al., 2015
Blood triglyceride		Positive	Vergès et al., 2012
Indirect VLDL-apoB100 FCR		Negative	Vergès et al., 2012
sdLDL	Atherosclerosis	Positive	Usui et al., 2009
SNPrs3758538	Obesity	Negative	Codoñer-Franch et al., 2016
ROS	Vascular injury, atherosclerosis	Positive	Wang et al., 2015
LVEF	Heart failure	Negative	Li et al., 2020
LVMI and LAD		Positive	von Jeinsen et al., 2018
TLR4 and MyD88		Positive	Gao et al., 2016
Carotid intima and plaque echogenicity	Coronary heart disease	Negative	Stakhneva et al., 2020
TTR	Amyloidosis	Positive	Suhr et al., 2015; Santos et al., 2016; Chan et al., 2017

*ANP, Atrial natriuretic peptide; GFR, glomerular filtration rate; VLDL, very low-density lipoproteins; sdLDL, small and dense low-density lipoprotein; ROS, reactive oxygen species; LVEF, left ventricular ejection fraction; LVMI, left ventricular mass index; LAD, left atrial internal diameter; TLR4, Toll-like receptor 4; MyD88, myeloid differentiation primary response gene 88; TTR, transthyretin protein.*

Obesity, one of the risk factors for atherosclerosis, can lead to increased blood triglyceride and cholesterol levels. The close correlation between visceral adiposity and cardiovascular disease has been previously demonstrated (Després and Lemieux, 2006). Won et al. (2012) found that RBP4 levels increased with the accumulation of visceral adiposity and were associated with risk factors for cardiovascular diseases (CVDs). RBP4 was more frequently expressed in visceral adipose tissue than in subcutaneous adipose tissue and was not affected by adiposity size, fat distribution, body fat percentage and other factors (Klöting et al., 2007). Serum RBP4 levels decreased by 25.5% in non-diabetic subjects after completing a 16-week weight loss program, and changes of RBP4 levels were significantly and positively associated with abdominal visceral fat loss, but not with total body fat loss or abdominal subcutaneous fat loss (Lee et al., 2008). In studies of genetic variants, different types of RBP4 single nucleotide polymorphisms (SNPs) were found to affect circulating RBP4 levels and were strongly associated with obesity, with the association of SNPrs3758538 with obesity being noteworthy, suggesting a possible predictive role of RBP4 gene variants on obesity risk (Tsutsumi et al., 1992). These studies suggest that RBP4 has a predictive value for visceral fat accumulation and that adipose tissue, as the main source of RBP4 secretion by the body (White and Kelly, 2001), suggests that RBP4 may be a key mediator of the increased risk of cardiovascular disease in obese patients.

### Retinol-Binding Protein 4 and Cardiovascular Diseases

#### Retinol-Binding Protein 4 and Vascular Injury

Oxidative stress-mediated changes promoted the development of cardiovascular disease (Massaeli and Pierce, 1995). It was found that RBP4 induces mitochondrial dysfunction and apoptosis, which in turn promotes vascular oxidative stress. RBP4 impaired mitochondrial number and integrity and reduced membrane potential by inducing reactive oxygen species (ROS) in mitochondria, and increased ROS and decreased ATP production affected normal endothelial cell. Increased cytochrome C release from mitochondria, increased Bax (pro-apoptotic protein) and decreased Bcl-2 were observed in arteries from RBP4 overexpressing (RBP4-Tg) mice, suggesting that RBP4 increased apoptosis of endothelial cell (Wang et al., 2015).

**FIGURE 1:**
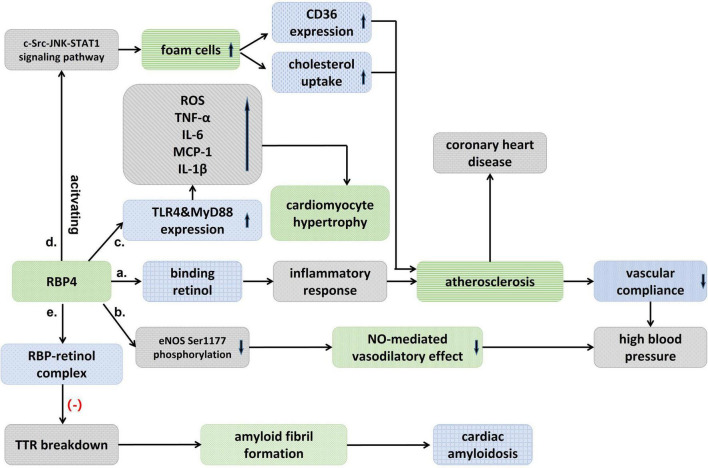
Schematic diagram showing an association between RBP4 and cardiovascular diseases. (a) Binding to retinol, RBP4 activates an inflammatory response that induces atherosclerosis and decreased vascular compliance, and thus raises blood pressure. (b) RBP4 could also result in high blood pressure by attenuating eNOS Ser1177 phosphorylation and then the nitric-oxide (NO)-mediated vasodilatory effect. (c) RBP4 stimulates TLR4 and MyD88 experssion, which significantly promotes the pro-inflammatory response and increases ROS, and then cause cardiomyocyte hypertrophy. (d) RBP4 promotes the formation of foam cells, which upregulates CD36 expression and cholesterol uptake, and atherosclerosis, thereby leading to coronary heart disease. (e) RBP-retinol complex could reduce TTR breakdown and then inhibit the formation of amyloid fibril and avoid cardiac amyloidosis.

Chronic vascular inflammation plays an important role in the development of atherosclerosis, and vascular dysfunction promotes plaque initiation and progression (Endemann and Schiffrin, 2004). RBP4 may be involved in the development of cardiovascular disease by inducing an inflammatory response. Norseen et al. (2012) found that RBP4 induced macrophage pro-inflammatory cytokine secretion and expression through activation of C-Jun N-terminal protein kinase (JNK) and Toll-like receptor 4 (TLR4)-dependent signaling pathways (Figure 1). Similarly, RBP4 was found to mediate vascular endothelial cell inflammatory responses *via* NADPH oxidase and NF-κB-dependent pathways (Farjo et al., 2012). In addition to endothelial cells, RBP4 also increases the proliferation of vascular smooth muscle cells through MAPK pathway and increases the risk of cardiovascular diseases (Li et al., 2015). the receptor and signaling pathways by which RBP4 acts with endothelial cells and VSMCs deserve to be explored in further studies, which may contribute to the understanding of the RBP4 and cardiovascular disease linkage.

#### Retinol-Binding Protein 4 and Hypertension

Hypertension is primarily a disease that results from genetic and environmental interactions, and its prevalence in the population is increasing year by year. It was found that blood RBP4 levels were significantly elevated in patients with untreated essential hypertension (Solini et al., 2009; Zachariah et al., 2016; Li et al., 2019) and significantly correlated with left ventricular diastolic function (Porcar-Almela et al., 2015; Li et al., 2019). RBP4 may be related to the left ventricular hypertrophy, and carotid intra-medial membrane thickness (IMT), suggesting that RBP4 may serve as a marker of vascular injury in hypertensive patients at early stage (Mansouri et al., 2012; Kraus et al., 2015). Significantly elevated levels of RBP4 were also found in patients with prehypertension, and RBP4 was independently associated with elevated diastolic and systolic blood pressure (Zhang et al., 2017). Insulin resistance (IR) has recently been suggested as a common pathophysiological basis for the development of type 2 diabetes and hypertension. Sasaki et al. (2020) found that the prevalence of hypertension increased with the degree of impaired glucose metabolism and elevated RBP4 may be involved in the development of hypertension by inducing IR. The mechanism of RBP4 in hypertension has not been clearly elucidated. Chiba et al. (2010) found that the binding of RBP4 to retinol activated inflammatory response that induced atherosclerosis, decreased vascular compliance and thus raised blood pressure. The correlation between RBP4 and arterial stiffness was also confirmed (Chondrou et al., 2020). It was found that blood pressure was reduced in RBP4 knockout (RBP-KO) mice and increased in RBP4 overexpressing (RBP-Tg) mice, while RBP-KO mice were protected from Ang-II-induced hypertension, confirming the presence of RBP4 as a risk factor in the development of hypertension (Kraus et al., 2015). The effect of RBP4 on blood pressure may be partly attributed to its effect on vascular endothelial function, as elevated RBP4 attenuates eNOS Ser1177 phosphorylation, which in turn attenuates the endothelium-dependent vasodilatory effect mediated by nitric oxide (Kraus et al., 2015; Figure 1).

Although the underlying mechanisms need to be further explored, more and more studies are now showing the close correlation between RBP4 and hypertension, and the monitoring of RBP4 may be a valuable indicator in determining early ventricular diastolic insufficiency and changes in vascular compliance in hypertensive patients. RBP4 may be a potential targeted molecule for preventing the progression of prehypertension and delaying ventricular remodeling.

#### Retinol-Binding Protein 4 and Heart Failure

Terminal B-type natriuretic peptide (NT-proBNP) is now known to be widely used as a diagnostic indicator of heart failure. Previous studies in elderly patients hospitalized with heart failure have shown that RBP4 did not correlate with NT-proBNP, and that changes of blood RBP4 levels are more likely attributable to the deterioration of renal function in patients with advanced heart failure, resulting in the accumulation of circulating adipokines (Majerczyk et al., 2018). It was found that alterations in RBP4 in patients with type 2 diabetes were also attributed to the changes of renal function (Henze et al., 2008). It seems to be no relationship between RBP4 and diagnosing of heart failure. But a subsequent prospective cohort study showed that in elderly patients with chronic heart failure, blood RBP4 levels were negatively correlated with left ventricular ejection fraction (LVEF) and positively correlated with NT-proBNP, and that serum RBP4 levels increased with decreasing cardiac function (Li et al., 2020; Table 1), In addition, the results of a 60-month follow-up suggested that blood RBP4 levels were positively correlated with adverse events in patients with chronic heart failure. The finding that RBP4 levels were positively correlated with left ventricular mass index (LVMI) and left atrial internal diameter (LAD) also suggests that RBP4 plays a role in the process of cardiac remodeling (von Jeinsen et al., 2018). The blood RBP4 levels in patients with advanced heart failure can be improved by implantation of a ventricular assist device (Chavarria et al., 2012). So RBP4 is a valuable diagnostic indicator of heart failure, and may be involved in the pathogenesis and development of heart failure.

Retinol-binding protein 4 is involved in the pathological process of heart failure through a variety of mechanisms. The elevation of RBP4 in patients with advanced heart failure may result from upregulation of RBP4 mRNA expression by IL-8 (Bobbert et al., 2009). In addition, RBP4 was found to cause cardiomyocyte hypertrophy, which may mediate a vicious cycle between insulin resistance and heart failure (Gao et al., 2016; von Jeinsen et al., 2018). RBP4 increased cell size, enhanced protein synthesis, and elevated the expression of hypertrophic markers including Anp, Bnp, and Myh7 in primary cardiomyocytes, but inhibition or knockdown of the TLR4/MyD88 pathway attenuated inflammatory and hypertrophic responses to RBP4 stimulation (Gao et al., 2016; Figure 1). Angiotensin II (Ang-II) also increases the expression of RBP4 in adipocytes, and the use of Ang-II receptor antagonists may eliminate this effect, which may explain another mechanism of the renin-angiotensin-aldosterone system (RASS) in exacerbating the deterioration of cardiac function (Gao et al., 2016). This needs to be explored in more clinical studies in the future.

#### Retinol-Binding Protein 4 and Coronary Heart Disease

Retinol-binding protein 4 levels in patients with coronary artery disease were found to be significantly higher than in the non-coronary artery disease (CAD) group (Ingelsson and Lind, 2009; Li et al., 2014; Liu et al., 2019) and positively correlated with the number of diseased vessels (Cubedo et al., 2014; Liu et al., 2015; Wessel et al., 2019), some clinical results have shown that serum RBP4 levels are reduced in patients with acute myocardial infarction, in male patients with familial hypercholesterolemia, a reduction in RBP4 has shown predictive significance for the possibility of ischemic events in the next 2 years, suggesting that RBP4 may be involved in the AMI (Cubedo et al., 2014). Similarly, Liu et al. (2015) found that patients with CAD with higher RBP4 had a concomitant increase in acute coronary syndrome (ACS) events in a 3-year follow-up (Table 1).

Previously, Mallat found that RBP did not provide additional predictive value compared to traditional risk factors in normal subjects (Mallat et al., 2009). But in a 16-year prospective case-control study (Sun et al., 2013), both full-length and total RBP4 levels were found to be strongly associated with the risk of coronary heart disease in women, with this association diminishing over time. Full-length RBP4 may exist as the most biologically active isoform of RBP4. In addition, increased values of both RBP4 and lipoprotein conjugate index (LCI) were found to be independent risk factors for ACS, and the combined test results of LCI and RBP4 values may serve as a potential indicator for the diagnosis of ACS (Wessel et al., 2019). RBP4 gene polymorphism was also found to be closely associated with coronary artery disease (Wan et al., 2014). Liu et al. (2017) found that RBP4 was localized in macrophage-rich foam cells, that RBP4 promotes the formation of macrophage-derived foam cells by activating the c-Src-JNK-STAT1 signaling pathway, which in turn upregulates CD36 expression and cholesterol uptake, and that RBP4 concentration is negatively correlated with carotid intima and plaque echogenicity (Stakhneva et al., 2020; Figure 1 and Table 1). The results suggested that RBP4 was involved in the progression of atherosclerosis. In conclusion, RBP4 involved in the pathophysiological process of coronary heart disease, as a risk factor, has shown valuable in predictor of coronary complexity and the occurrence of adverse cardiovascular events in patients with CAD. RBP4 is expected to be a new biological indicator of coronary heart disease, and a clinical risk factor of coronary heart disease.

#### Retinol-Binding Protein 4 and Cardiac Amyloidosis

Cardiac amyloidosis (CA) is an accumulation of insoluble fibrous deposits composed of abnormally folded protein molecules in the myocardial interstitial, which mainly manifests clinically as cardiac insufficiency, various arrhythmias, and angina pectoris, among which the transthyretin amyloidosis (ATTR) type is more common in clinical work. It was found that blood RBP4 levels in patients with transthyretin amyloidosis were significantly lower than in controls (Arvanitis et al., 2017a,b). Chan et al. (2017) further found that RBP4 was significantly decreased in mutant myocardial amyloidosis (ATTRm) compared to controls, but not in wild-type myocardial amyloidosis (ATTRwt) by using blood proteomics analysis. The reduced level of serum TTR was closely correlated with the reduced level of RBP4 (Suhr et al., 2015). The lower levels of RBP4 paralleled to serum TTR levels, which may be related to the fact that TTR acts as a transporter protein to bind the RBP4-retinol complex and thus reduces renal excretion of RBP4 (Hamilton and Benson, 2001; Table 1).

Retinol-binding protein 4 has been found to have anti-amyloidogenic properties, and the decrease of RBP4 alleviate the formation of the RBP4-retinol complex and attenuates its role in stabilizing the TTR tetrameric structure (White and Kelly, 2001; Santos et al., 2016). *In vitro* assays have shown that the RBP-retinol complex (holoRBP) inhibits the rate of amyloid fibril formation in a concentration-dependent manner (White and Kelly, 2001). holoRBP at physiological concentrations slowed down the rate of TTR breakdown approximately sixfold compared to TTR breakdown alone (Hyung et al., 2010). RBP4 concentrations higher than or equal to 50 μg/mL were found to be up to 100% sensitive for the diagnosis of ATTRV122I amyloidosis, although its specificity decreased to 38%, suggesting that RBP4 could provide 100% negative predictive value when used to rule out ATTRV122I amyloidosis (Arvanitis et al., 2017a; Table 1). The diagnostic and predictive value of RBP4 in ATTR amyloidosis needs to be confirmed in larger and more pathologically diverse cohort studies.

## Summary

As an adipokine, RBP4 has shown a close association with dyslipidemia, obesity, and vascular impairment. RBP4 also derives exclusively from hepatocytes, but liver-secreted RBP4 does not impair glucose homeostasis (Fedders et al., 2018). In addition, RBP4 has shown promising value for cardiovascular disease diagnosis and treatment, such as predicting the risk of hypertension and coronary heart disease in the general population, and assessing the prognosis of patients with coronary heart disease and heart failure, etc. RBP4 is expected to be a new biomarker for cardiovascular disease in the future. The study of RBP4 antagonists may also be a new therapeutic agent for cardiovascular diseases.

## Author Contributions

XG conceived the study and designed the study protocol. YJ and JS conducted the literature review and drafted the manuscript. TS reviewed the manuscript for intellectual content, made revisions as needed. All authors contributed to the article and approved the submitted version.

## Conflict of Interest

The authors declare that the research was conducted in the absence of any commercial or financial relationships that could be construed as a potential conflict of interest.

## Publisher’s Note

All claims expressed in this article are solely those of the authors and do not necessarily represent those of their affiliated organizations, or those of the publisher, the editors and the reviewers. Any product that may be evaluated in this article, or claim that may be made by its manufacturer, is not guaranteed or endorsed by the publisher.
